# Evaluation of the Accuracy of Point-of-Care Urine Chloride Measured via Strip Test in Patients with Heart Failure

**DOI:** 10.3390/biomedicines12112473

**Published:** 2024-10-28

**Authors:** Mateusz Guzik, Berenika Jankowiak, Piotr Ponikowski, Jan Biegus

**Affiliations:** Institute of Heart Diseases, Faculty of Medicine, Wroclaw Medical University, Borowska 213, 50-556 Wroclaw, Poland

**Keywords:** heart failure, strip test, urine chloride, urine sodium, self-monitoring

## Abstract

Background: In clinical practice, patient self-monitoring is crucial in achieving therapeutic goals in various diseases. In heart failure (HF), it is particularly important due to the increasing role of urine composition. Therefore, we proposed this study to assess the accuracy of urine chloride (*uCl*^−^) assessment via strip test in relation to chloride and sodium (uNa^+^) measurements in a gold-standard laboratory method. Methods: Urine samples were collected before administering morning medications. Afterwards, they were analyzed concurrently using the strip test and gold-standard laboratory method. Results: The study cohort comprised 66 patients (82% male, mean age 68 ± 12 years), of whom 65% were diagnosed with HF and 35% without HF. Across the entire cohort, a strong correlation was observed between *uCl*^−^ measured by both methods (r = 0.85; *p* < 0.001). However, the strip test was found to underestimate *uCl*^−^ relative to the laboratory measurements (mean difference of 18 mmol/L). Furthermore, strong correlations were observed between the methods among patients with HF and without HF (r = 0.88 vs. r = 0.71, respectively; *p* < 0.001 for both), where they presented similar relationship patterns. Interestingly, in patients with a low glomerular filtration rate (eGFR ≤ 60 mL/min/1.73 m^2^), the correlation between both methods was greater compared to those with high eGFR (>60 mL/min/1.73 m^2^) (r = 0.94 vs. r = 0.76, respectively; *p* < 0.001 for both). The relationship between *uCl*^−^ from the strip test and uNa^+^ from the laboratory measurement was weaker than for *uCl*^−^, but it was significant. Conclusions: These findings suggest that point-of-care strip tests for assessing urinary chloride demonstrate high accuracy and potential utility, particularly in patients with reduced eGFR.

## 1. Introduction

The effective treatment and management of patients with chronic diseases is only possible when they actively participate in the process. Therefore, awareness of their disease, condition, and the importance of self-assessment is crucial. In addition to easily obtainable clinical parameters like body weight and blood pressure, there is an opportunity to extend self-monitoring to include certain hemodynamic and biochemical analyses [1,2]. Among the various parameters recommended for monitoring in clinical conditions, assessing urine composition can be helpful in certain diseases, especially heart failure (HF). It is well established that routine urine assessment in HF serves as an indicator of diuretic response [3,4,5,6,7]. Evidence suggests that, nowadays, in acute HF, diuretic treatment should be guided by urine sodium concentration [5,8,9,10]. However, due to the difficulty and impracticality of regular/daily urine biochemical parameter assessment, there are no data on such a method for chronic HF outpatient monitoring. Nevertheless, numerous studies have shown that low concentrations of chloride and sodium in the urine are associated not only with a poor diuretic response, but also with worse long-term outcomes [11,12,13]. This is reasoned in pathophysiology to be because chloride concentration plays a significant role in the regulation of renal blood flow, glomerular filtration, and ion reabsorption/secretion mechanisms in relation to neurohormonal activity [12,14,15,16,17]. Importantly, this could contribute to the development of cardiorenal syndrome, which might result in the deterioration of heart and kidney function, leading to impaired glomerular filtration, reduced urine production, and diminished response to loop diuretics [10]. This could limit the effectiveness of decongestive therapy in acute HF and the maintenance of the euvolemic state in chronic HF [12,14]. Additionally, the administration of high doses of diuretics in outpatients could cause dehydration, thirst, and further kidney injury, which could result in diminished sodium and chloride excretion [18,19,20,21]. Therefore, a long-term approach involving strict monitoring of critical parameters, such as chloride or sodium excretion, and adjusting the patient’s treatment accordingly may be justified. Strip tests that allow for the evaluation of urine chloride concentration have been evaluated in patients with cerebral salt-wasting syndrome, those on home parenteral nutrition, and those with hyponatremia or dehydration. The results were promising. Thus, our aim was to investigate the chloride concentration in urine using a strip test (as a point-of-care examination) to determine the accuracy of such an approach (in comparison to the gold-standard laboratory method). Additionally, due to the absence of strip tests for urine sodium assessment, we evaluated the relationship between urine chloride (assessed via strip test) and urine sodium laboratory assessments, as the currently most evidenced urine ion.

## 2. Materials and Methods

The study was a prospective, observational, single-center study conducted among patients hospitalized between April and May 2024 in the Institute of Heart Diseases in Wrocław, Poland. The participants included patients with established diagnoses of chronic heart failure, with stable symptoms over time, as well as patients without a diagnosis of heart failure. Basic clinical, laboratory, and echocardiographic data were collected from each participant. The participants were asked to provide a urine sample for biochemical assessment. First, morning urine samples (20 mL) from the first day of hospitalization were collected from patients before they took their morning medications. Each obtained sample was divided into two identical, sterile containers, where both contained 10 mL of urine, to undergo independent assessments. The samples were assessed in the laboratory to measure the chloride and sodium levels in the urine. Simultaneously, the urine was tested using a strip test (Hach, Quantab). All urine samples were assessed by one single physician to simulate the condition in which the patient would self-assess the urine sample.

According to the instructions, the strip test result was taken once the strip indicated the end of the assessment (the indicator at the top of the strip turned black). All strip test results were evaluated by one investigator, blinded to the laboratory measurement results at the time of evaluation. The values indicated in the strip test (mm) were converted to mmol/L using the scale provided in the test specification. The results obtained from the laboratory assessments and the strip tests were compared using the statistical methods described below. Due to the local laboratory’s low limit of sodium and chloride measurement, patients with lower than 20 mmol/L sodium and/or chloride established in the strip test were excluded from the study. Additionally, patients whose tests were not completed according to the test instructions were excluded. 

In terms of statistical analysis, due to the lack of prior studies, no evidence-based a priori assumptions regarding correlations and sample size could be made. However, based on power analysis (power: 0.80; alpha: 0.05; assumed correlation coefficient: 0.5), the required sample size was estimated at 29 patients. The quantitative variables were analyzed for normality using the Shapiro–Wilk test. Variables with normal distribution are presented as mean ± standard deviation (SD), while those that did not follow a normal distribution are presented as median [interquartile range (IQR)]. The homogeneity of variance was tested by the Levene test. The differences between subsequent variables with normal distribution were evaluated using the *t*-test. Welch’s correction was applied when appropriate. Variables with a distribution other than normal were tested using the Mann–Whitney U test. Differences between qualitative variables were examined using the Chi-Square test. The Pearson correlation test was used to evaluate correlations between values. The Bland–Altman plot was performed to assess the agreement between two measurement methods by plotting the differences between them against their average. Points close to the mean difference indicate good agreement, while those further from the mean suggest discrepancies. The limits of agreement in the Bland–Altman plots were calculated based on the mean difference of measurements: mean difference ± 1.96 standard deviations. Post hoc tests’ power was checked. A graphical presentation of the results was made using GraphPad Prism. The statistical analysis was performed in Statistica 13.

## 3. Results

### 3.1. Study Population Characteristics

The analysis included 66 patients, predominantly male (79%), with a mean age of 68 ± 12 years. Among the participants, 65% had been diagnosed with heart failure. The median left ventricle ejection fraction (LVEF) was 50 [30–57]%, and the NT-proBNP concentration was 1583 [274–6480] pg/mL. Additionally, 51% of chronic heart failure patients were taking diuretics, with a median furosemide (or equivalent) dose of 20 [0–60] mg. 

The summarized characteristics of the study population are presented in Table 1.

### 3.2. The Relationship Between the Value Taken from the Strip Test and the Corresponding Chloride Concentration

**Figure 1:**
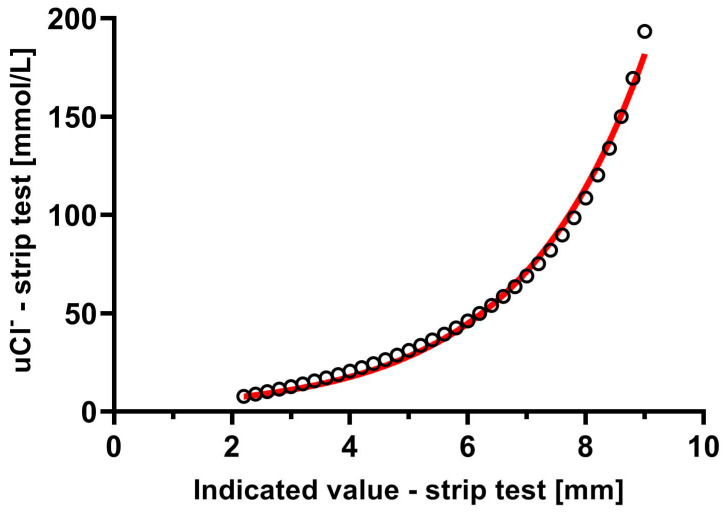
The relationship between corresponding values: the value taken from strip test and corresponding urine chloride concentration according to test specification.

The pattern between the value taken from the strip test and the corresponding urine chloride is an exponential shape. It was presented in Figure 1. The equation of the best-fit curve is as follows:

(1) $$uCl=3.33\times e^{0.44\times D}$$

*uCl*^−^—Urine chloride concentration.
*D*—Value taken from strip test.

To examine the fit of the curve describing the relationship between the presented values, the relationship was linearized by the logarithmizing the equation:

ln (*uCl*) =ln 3.33 × 0.44 × *D* (2)

*uCl*^−^—Urine chloride concentration.
*D*—Value taken from strip test.

For these data R^2^ value was 0.998.

### 3.3. The Relationship Between Urine Chloride Measured in the Laboratory and in the Strip Test

In general, the relationship pattern is linear (with correlation r = 0.85; *p* < 0.001), but the results are dispersed over 40 mmol/L *uCl*^−^ with the strip test, especially for higher concentrations of *uCl*. Nevertheless, discrepancies resulted from the underestimation of *uCl* in the strip test (laboratory-assessed *uCl* was, in general, equal to or higher than that measured by the strip test).

**Figure 2:**
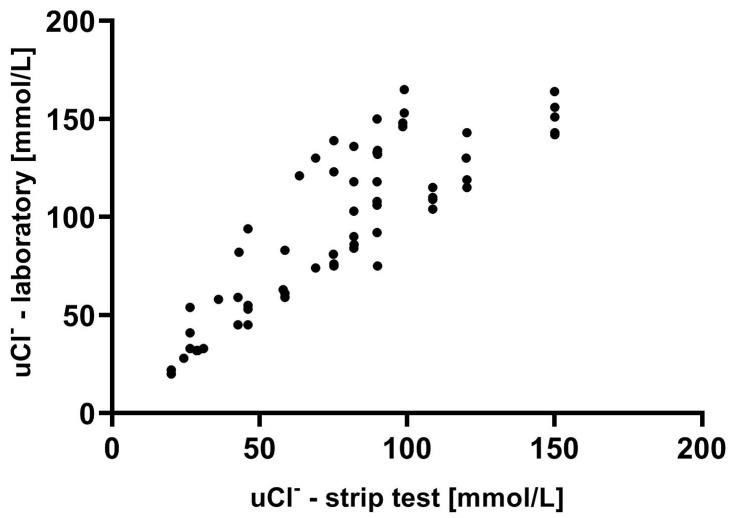
Relationship between urine chloride measured in the laboratory and in strip test across the entire cohort.

Based on a linear regression model, the function of dependence between *uCl* measured in the laboratory and obtained in the strip test was established (Figure 2).

This equation was as follows:

(*uCl*-lab) = 1 × (*uCl*-strip) + 17.94; *p* < 0.001 (3)

#### 3.3.1. The Relationship Between Urine Chloride Measured in the Laboratory and the Strip Test in HF vs. No-HF Patients

**Figure 3:**
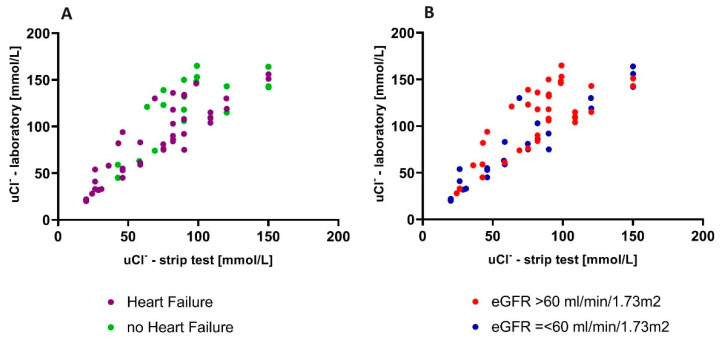
The relationship between urine chloride measured in the laboratory and in strip test in patients with HF vs. without HF (**A**) and with low vs. high eGFR (**B**).

In terms of the accuracy of the *uCl*^−^ measured in the strip test (*uCl*-strip) and the *uCl*^−^ or uNa^+^ evaluated in a local laboratory (*uCl*-lab and uNa-lab, respectively), the individuals with HF presented a stronger correlation between the urine chloride obtained in the strip test and in the laboratory (r = 0.88 in chronic HF and r = 0.71 in no-HF patients, *p* < 0.001 for both). The linear regression models were as follows: (*uCl*-lab) = 0.98 × (*uCl*-strip) + 15.77 (*p* < 0.001) in HF patients and (*uCl*-lab) = 0.77 × (*uCl*-strip) + 47.91 (*p* < 0.001) in no-HF individuals. This relationships are presented in Figure 3A. The subgroups’ characteristics and the accuracy of fit between the laboratory and strip-established ion concentrations are presented in the Supplementary Materials.

#### 3.3.2. The Relationship Between Urine Chloride Measured in the Laboratory and in the Strip Test in Low-e-GFR vs. High-eGFR Patients

We divided the whole study cohort according to eGFR (eGFR > 60 mL/min/m^2^ (named: “low eGFR”) and eGFR ≤ 60 mL/min/m^2^ (“high eGFR”)). A well-fitted linear pattern was observed for patients with low eGFR (N = 26) (r = 0.94, *p* < 0.001), with a linear regression equation as follows: ((*uCl*-lab) = 0.96 × (*uCl*-strip) + 11.22; *p* < 0.001). Those with high eGFR (N = 40) also presented a linear correlation between the values obtained in both tests (r = 0.74; *p* < 0.001), but this was weaker than for the low-e-GFR group. The linear regression equation was as follows: (*uCl*-lab) = 0.92 × (*uCl*-strip) + 28.20; *p* < 0.001). This relationship is presented graphically in Figure 3B. The characteristics of the subgroups and the accuracy of fit between the ion concentrations measured in the laboratory and those established by strip testing, shown on Bland–Altman plots, are provided in the Supplementary Materials.

### 3.4. The Relationship Between Urine Sodium Measured in the Laboratory and Urine Chloride in Strip Tests

**Figure 4:**
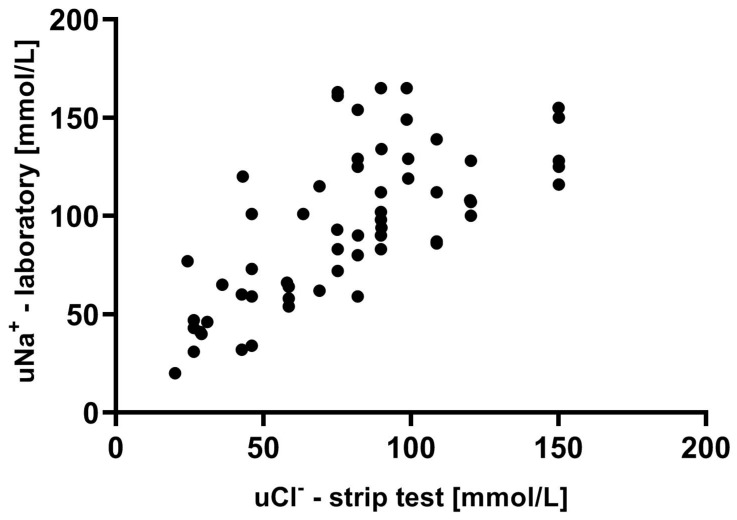
The relationship between urine sodium concentration measured in the laboratory and urine chloride obtained in strip tests for the entire cohort.

The pattern of the relationship between uNa^+^ laboratory measurements and *uCl*^−^ from the strip tests was weaker than in the case of urine chloride (r = 0.71; *p* < 0.001), with the following regression equation: (uNa-lab)= 0.81 × (*uCl*-strip) + 30.11; *p* < 0.001 (Figure 4).

**Figure 5:**
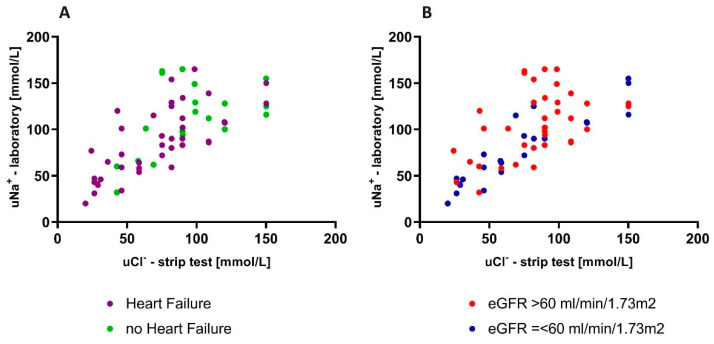
The relationship between urine sodium concentration measured in laboratory and urine chloride obtained in strip test in patients with HF vs. without HF (**A**) and low vs. high eGFR (**B**).

The correlation between both measured values in HF patients was r = 0.71 (*p* < 0.001), and in the no-HF subgroup r = 0.52 (*p* = 0.02). These linear relationships can be described by the following equations: (uNa-lab) = 0.80 × (*uCl*-strip) + 30.68; *p* < 0.001 and (uNa-lab) = 0.59 × (*uCl*-strip) + 56.16; *p* = 0.02), respectively. This is presented in Figure 5A. 

The correlation between uNa-lab and *uCl*-strip was stronger for those with low eGFR (r = 0.91; *p* < 0.001) than for high eGFR (r = 0.49, *p* < 0.01), as described by the following equations: (uNa-lab) = 0.85 × (*uCl*-strip) + 16.94 (*p* < 0.001) and (uNa-lab) = 0.65 × (*uCl*-strip) + 49.93 (*p* < 0.001), respectively. The patterns of these relationships are presented in Figure 5B.

### 3.5. Graphical Presentation of Agreement Between Urine Chloride and Sodium Measured in Laboratory and Urine Chloride Measured in Strip Tests

**Figure 6:**
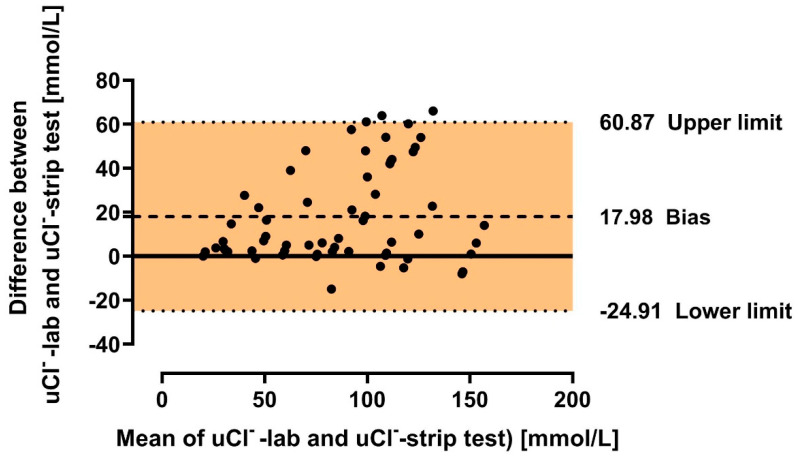
Bland–Altman plot for agreement of urine chloride concentration measured by strip tests and urine chloride measured in the laboratory in the general cohort. Legend: *uCl*^−^—urine chloride; Bias—mean difference between strip test and laboratory test results; Lower limit—mean difference-1.96 × standard deviation; Upper limit—mean difference + 1.96 × standard deviation.

**Figure 7:**
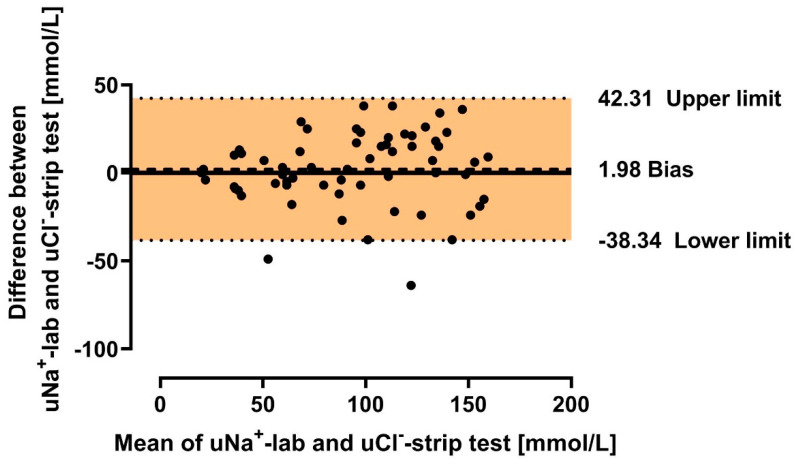
Bland–Altman plot for agreement of urine chloride concentration measured by strip test and urine sodium measured in the laboratory in the general cohort. Legend: *uCl*^−^—urine chloride; uNa^+^—urine sodium; Bias—mean difference between strip test and laboratory test results; Lower limit—mean difference-1.96 × standard deviation; Upper limit—mean difference + 1.96 × standard deviation.

The degree of agreement between urine chloride concentration obtained in strip-test and urine chloride and sodium obtained in the laboratory (gold standard) method were presented in Bland-Altmann plots (Figure 6 and Figure 7). The subgroups analyses were presented in Supplementary Materials.

### 3.6. Sensitivity, Specificity, and Positive and Negative Predictive Values of uCl^−^ Strip Test for an Indication of Poor Diuretic Response

In chronic heart failure, there is no widely established cut-off point for uNa^+^ and/or *uCl*^−^ indicating a poor treatment response. However, we conducted a simulation assuming a cut-off point of 70 mmol/L for both uNa^+^ and *uCl*^−^, established in patients with acute heart failure, to define poor diuretic response (<70 mmol/L). We then calculated the parameters (sensitivity, specificity, and positive and negative predictive values) for the *uCl*^−^ strip test (<70 mmol/L) for the indication of poor treatment response, predefined using the above the cut-off point. The obtained sensitivity, specificity, and positive and negative predictive values were satisfactory (Table 2).

### 3.7. Test Power Analysis

Based on the obtained correlation coefficients and cohort sizes, and assuming an alpha level of 0.05, the test power was approximately 1.0 for the entire study cohort when divided into the above subgroups. However, in the analysis of *uCl*-strip and uNa-lab in the non-heart failure population, the test power was 0.73.

### 3.8. Issues of Measurement and Notation Bias

**Figure 8:**
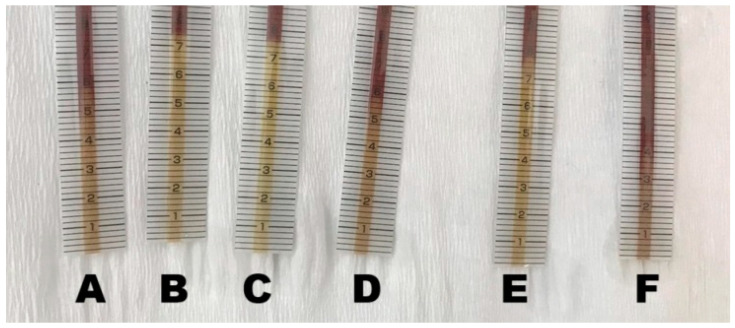
Examples of different patterns of urine chloride markers in the strips ((**A**)–(**F**)).

Despite the validated method of chloride evaluation via strip test, which has been known for many years, some issues were found during the study processing. The first was the inconsistency in the pattern of the top borders of the concentration indicators across measurements. For instance, instead of a flat line, in some cases a triangle shape is seen with no clearly visible tip (Figure 8D–F). This may cause the bias of underestimation or overestimation (depending on the user assessment). For comparison, the well-visible markers were shown in Figure 8B,C. Other tests do not have sharply defined borders, but instead, their color gradually changes. An additional issue is the different times taken for patients to fill the strip and show the proper results. There were some technical issues in some cases; for some patients, the tests did not reach the marker indicating the end of the assessment (despite a long waiting time), which did not allow us to classify these tests as reliable.

## 4. Discussion

The presented study shows a high correlation between the urine chloride evaluated in strip tests and urine chloride and/or sodium assessment using the gold-standard laboratory method. The results of this study reveal several significant implications. 

The first one is the fact that the concentration of *uCl*^−^ was underestimated by the measurement achieved via the strip tests in comparison to the laboratory assessments. This was confirmed by the mathematical analysis of the relationship between the variables. Note that the values of *uCl*-lab are higher than *uCl*-strip for the entire population and subsequent subgroups (see equations and plots in results). Furthermore, the pattern of changes between *uCl*^−^ or uNa^+^ from the laboratory tests and *uCl*^−^ obtained in the strip tests was flatter for those without HF or with high eGFR. Only in the case of urine chloride comparison regarding eGFR were the patterns of the relationship almost parallel. This raises some doubt as to whether using such tests in outpatient care in the general population as the sole marker influencing decisions regarding a possible increase in doses of diuretics might lead to overdiagnosis by the patient of conditions threatening the decompensation of chronic heart failure. 

A few studies conducted using strip tests for chloride assessment have shown different degrees of correlation between the test results and laboratory measurements. These results differ between studies, showing the highest agreement in healthy individuals and slightly lower agreement in populations of patients with intestinal and neurological disorders (suspected cerebral salt-wasting). Nonetheless, there are no studies providing insight into the usefulness of chloride strip tests in heart failure [22,23]. Heart failure patients take diuretics and other drugs that chronically influence neurohormonal activity, which is overactivated in this condition. Consequently, kidney function and diuretic response are affected by these factors, leading to significant differences among heart failure patients and other tested populations [14,24,25,26,27,28].

Another issue is the significantly better fit of the values measured by the strip test with those obtained in laboratory assessments in patients with low eGFR compared to patients with high eGFR values. The results indicate that the model based on these values explains approximately 90% of the obtained measurements. This gives us basis to suspect that the use of strip tests in certain groups (e.g., patients with low eGFR) could be successfully applied in outpatient management, indicating the high reliability of the measurements made by educated, aware patients.

Thirdly, the chloride concentration measured using the strip test showed that there is also a significant relationship with the sodium concentration in urine (assessed in the laboratory). This relationship was inherently weaker than that for chlorides, but also showed a profile similar to the relationship between the chloride concentrations from the strip test and the laboratory measurements. Again, a significantly stronger relationship was observed in the groups of patients with HF and low eGFR. Studies have demonstrated the significant relationships of chloride established in strip tests in relation to natriuresis. Their results are similar to those obtained in our study cohort despite the different populations included in the analysis [23]. It is noteworthy that the subsequent analyses that were conducted were characterized by satisfactory test power.

Another, fourth, important topic is the various issues that may affect measurement bias, consequently impacting the checked/indicated value of the test. Note that the higher the value indicated in the strip test (on an interval scale), the greater the risk of measurement error due to the exponential relationship between this value and its assigned concentration. Hence, the lower the chloride concentration, the lesser the potential impact of measurement error on the obtained value (and potential further clinical decisions). For example, the difference between a strip test result of 4.6 and 4.8 translates to a difference in chloride concentration of 29 to 31 mmol/L, while between a reading of 8.6 and 8.8, the increase in chloride concentration is significantly greater—150 to 169 mmol/L! This is reflected in the Bland–Altman plots, where the difference between the values obtained from the strip tests and the laboratory tests increases with higher mean test values (Figure 6 and Figure 7, and Supplementary Materials). Additionally, the issue with indicator boundaries (e.g., poor visibility or results falling between two indicator lines), as discussed in a separate section, may contribute to measurement imprecision, especially at increasing strip test values. In the study, we deliberately decided that a single investigator would read the tests to simulate the situation where a patient would need to interpret the test result during self-assessment. A consensus evaluation by multiple researchers was not conducted, as this could potentially fail to reflect the bias associated with individual assessment. An action that could be useful in the future, limiting human factors’ impact on obtaining strip test results, could be the use of devices utilizing the digital analysis of ion concentrations in urine. Unfortunately, these devices are relatively more expensive and thus less accessible to patients. Additionally, no studies have proven the superiority of digital chloride/sodium assessment in urine compared to strip tests in either the heart failure population or other groups. Nevertheless, the dynamic development of digital technologies (especially artificial intelligence) may provide a tool in the future for the low-cost, simple-in-use, and reliable evaluation of biochemical body fluids. It should be highlighted that similar strip tests have been tested in the context of predicting 24 h urinary sodium excretion based on various protocols for collecting and assessing single urine samples. In these studies, flame photometry, among other methods, was used to assess sodium concentration as a reference method. The results were consistent with the strip test results [29]. Additionally, various strip tests have also been examined for their agreement with reference results, showing general consistency in three out of four tested systems, although each had its own problems—difficulty in reading the result, the need to dilute urine samples due to exceeding the test range, etc. [30].

Taking into account the presented results and discussion, the clinical applicability of this study lies in the potential use of strip tests for outpatient self-monitoring, or as a point-of-care tool for inpatients. This could become an integral element in the development of remote monitoring for patients with heart failure. In the future, such measurements could play a crucial role in home diuretic titration, which is particularly important given the association between higher doses of loop diuretics and worsening outcomes [13,31,32]. 

## 5. Conclusions

Our study shows that strip tests appear to be a highly accurate tool for *uCl*^−^ assessment, with a high correlation with the gold standard (laboratory assessment). There was a relationship between gold-standard laboratory urine chloride, sodium, and strip test chloride measurements. Nevertheless, the results were not perfectly matched. Moreover, promising data obtained in patients with low eGFR indicate the potential usefulness of chloride strip tests, with high reliability in specific populations with chronic heart failure. However, due to the limitations (relatively low sample size) and character (single-center study) of our work, multicenter, randomized studies based on sequential spot urine chloride and/or sodium assessments are necessary to extrapolate the abovementioned results onto the broad heart failure population. Additionally, dynamic developments in computer-based assessments (e.g., artificial intelligence) may be useful in the future to check strip test results.

## 6. Limitations

This is a single-center, observational study conducted on a relatively small population (but large enough to obtain significant testing power). This limits the generalization of the results and does not allow us to present causality. Moreover, there are no widely established urine monitoring tools to compare with the strip test in a cohort of heart failure patients. 

The strip test results were assessed by a single dedicated physician, which could introduce reading bias (especially due to different patterns of result indications—see “Issues of measurement and notation bias”). Moreover, the ion concentration agreement between the strip tests and the laboratory “gold standard” was based on a single assessment. Multiple measurements using average results could potentially provide greater consistency between tests. The study design, however, deliberately promoted single-user reading to simulate the real-life scenario in which the patient would read the strip test by themself (not knowing how far individual results deviate from the actual value). However, as mentioned above, this design is related to some limitations. In clinical practice, some risk of measurement error (and variation) would be acceptable if it did not significantly affect the decision-making process. As we did not store the urine samples or strip tests during the study, we cannot compare them further with different utilities or re-assess the strip tests using multiple investigators.

## Figures and Tables

**Table 1:** General characteristics of population.

Parameter	Value
Age (years)	68 ± 12
Sex—male (N; %)	52 (79%)
HR (beats/min)	71 [63–80]
SBP (mmHg)	123 ± 18
DBP (mmHg)	75 ± 11
MAP (mmHg)	91 ± 11
Hgb (g/dL)	12.8 ± 2
Leukocytes (10^3^/uL)	7.47 [5.81–9.85]
Creatinine (mg/dL)	1.08 [0.84–1.68]
eGFR (mL/min/1.73 m^2^)	70 ± 31
Urea (mg/dL)	46 [32–73]
NT-proBNP (pg/mL)	1 583 [274–6480]
Serum Na^+^ (mmol/L)	141 ± 3
Serum K^+^ (mmol/L)	4.4 ± 0.5
LVEF (%)	50 [30–57]
TAPSE (mm)	21 ± 5
Arterial hypertension (N; %)	49 (74%)
Diabetes mellitus (N; %)	35 (53%)
Amyloidosis (confirmed or suspected) (N; %)	2 (3%)
Chronic kidney disease (N; %)	23 (35%)
Chronic diuretics administration (N; %)	34 (52%)
Chronically administered loop diuretic dose (furosemide dose equivalent) (mg)	20 [0–60]
SGLT-2 inhibitors (N; %)	33 (48%)
B-blocker (N; %)	55 (83%)
ACEI/ARB (N; %)	31 (47%)
ARNI (N; %)	8 (12%)
MRA (N; %)	31 (47%)
Acetazolamide (N; %)	0 (0%)
Thiazide diuretics (N; %)	3 (5%)

**Table 2:** Parameters of *uCl*^−^ strip indication for poor treatment response (cut-offs: uNa-lab < 70 mmol/L and *uCl*-strip < 70 mmol/L).

Parameter	Sensitivity	Specificity	Positive Predictive Value	Negative Predictive Value
uNa-lab < 70 mmol/L	94.7%	85.7%	75.0%	97.3%
*uCl*-lab < 70 mmol/L	100%	86.0%	75.0%	100%

## Data Availability

Data can be shared upon explicit request.

## Supplementary Materials

The following supporting information can be downloaded at: https://www.mdpi.com/article/10.3390/biomedicines12112473/s1.
