# Supplementary material for: Evaluation of the Accuracy of Point-of-Care Urine Chloride Measured via Strip Test in Patients with Heart Failure
Source: Biomedicines. 2024 Oct 28;12(11):2473. doi: 10.3390/biomedicines12112473 (PMC11591833; doi:10.3390/biomedicines12112473)

Supplementary materials

Supplementary Table S1. *Comparison between patients with HF and without HF.*

Parameter	Value		p
	HF (N=43 (65%))	No HF (N=23 (35%))	
Age (years)	68±12	65±14	0.37
Sex – male (N;%)	39 (91%)	13 (65%)	0.01
HR (beats/min)	75 [66-81]	69 [60-80]	0.27
SBP (mmHg)	122±21	125±12	0.25
DBP (mmHg)	75±11	76±9	0.54
MAP (mmHg)	76±17	92±8	0.32
Hgb (g/dl)	12.7±2.2	13.0±1.6	0.51
Leukocytes (10^3/ul)	8.05 [6.53-10.2]	6.68 [5.39-9.33]	0.14
Creatinine (mg/dl)	1.25 [0.89-1.80]	0.98 [0.81-1.19]	0.04
eGFR (ml/min/1,73m^2)	66±35	77±20	0.07
Urea (mg/dl)	55 [40-82]	37 [26-44]	<0.01
NT-proBNP (pg/ml)	3362 [1371-9264]	266 [158-900]	<0.001
Serum Na^+ (mmol/l)	140.9±3.1	140.6±2.3	0.71
Serum K^+ (mmol/l)	4.4±0.6	4.4±0.4	0.86
LVEF (%)	34 [27-48]	57 [54-60]	<0.01
TAPSE (mm)	19±5	25±4	0.97
Arterial hypertension (N;%)	32 (74%)	17 (74%)	0.03
Diabetes Mellitus (N;%)	26 (60%)	9 (39%)	0.07
Amyloidosis (confirmed or suspected) (N;%)	2 (5%)	0	0.76
Chronic diuretics administration (N;%)	31 (72%)	3 (13%)	<0.001

Chronically administered loop diuretic dose (furosemide dose equivalent) (mg)	40 [20-80]	0 [0-0]	<0.001
SGLT-2 inhibitors (N;%)	31 (72%)	2 (9%)	0.13
B-blocker (N;%)	40 (93%)	15 (65%)	0.11
ACEI/ARB (N;%)	21 (49%)	10 (43%)	0.07
ARNI (N;%)	8 (19%)	0 (0%)	0.51
MRA (N;%)	27 (63%)	4 (17%)	0.76
Acetazolamide (N;%)	0 (0%)	0 (0%)	1.00
Thiazide diuretics (N;%)	2 (5%)	1 (4%)	0.54

Supplementary Figure S1. *Bland-Altmann plot for agreement of urine chloride concentration measured by strip-test and urine chloride measured in the laboratory in patients with HF. Legend: uCl$^-$ - urine chloride, Bias – mean difference between strip test and laboratory test results, Lower limit – mean difference-1.96 × standard deviation, Upper limit – mean difference+1.96 × standard deviation*

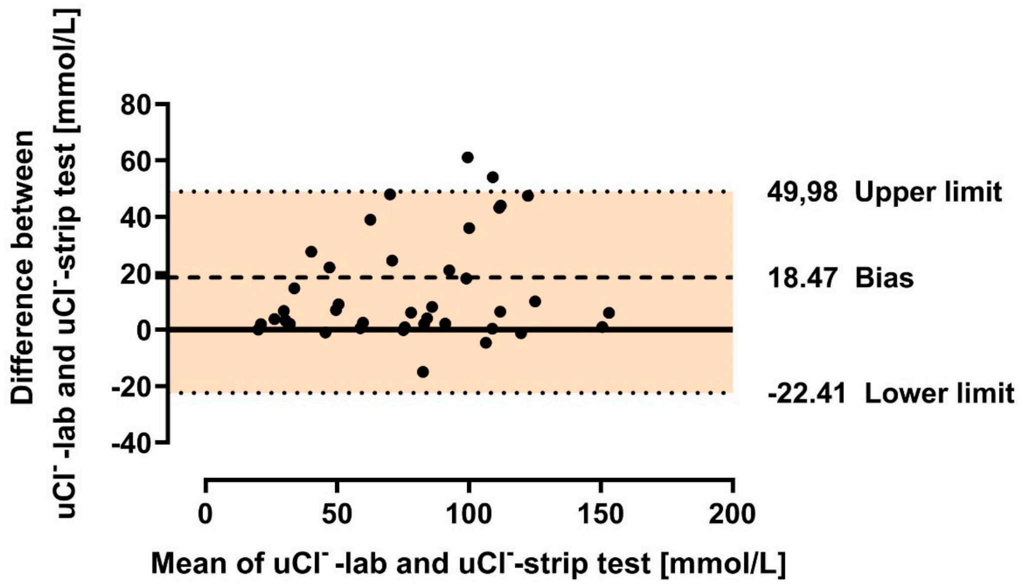

Supplementary Figure S2. *Bland-Altmann plot for agreement of urine chloride concentration measured by strip-test and urine chloride measured in the laboratory in patients with no HF. Legend: uCl$^-$ - urine chloride, Bias – mean difference between strip test and laboratory test results, Lower limit*

– mean difference-1.96 × standard deviation, Upper limit – mean difference+1.96 × standard deviation.

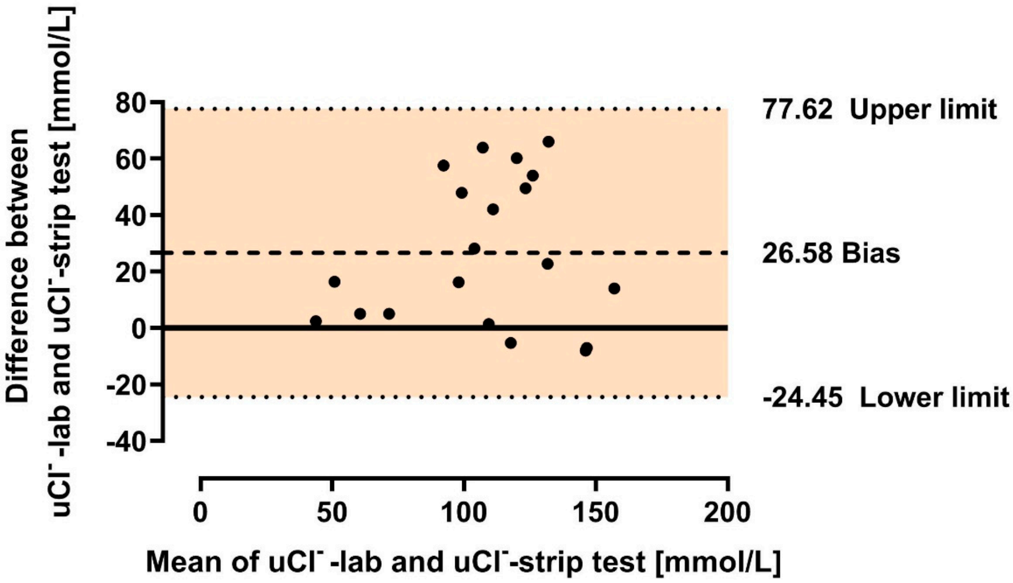

Supplementary Figure S3. *Bland-Altmann plot for agreement of urine chloride concentration measured by strip-test and urine sodium measured in the laboratory in patients with HF. Legend: uCl^- - urine chloride, uNa^+ - urine sodium, Bias – mean difference between strip test and laboratory test results, Lower limit – mean difference-1.96 × standard deviation, Upper limit – mean difference+1.96 × standard deviation.*

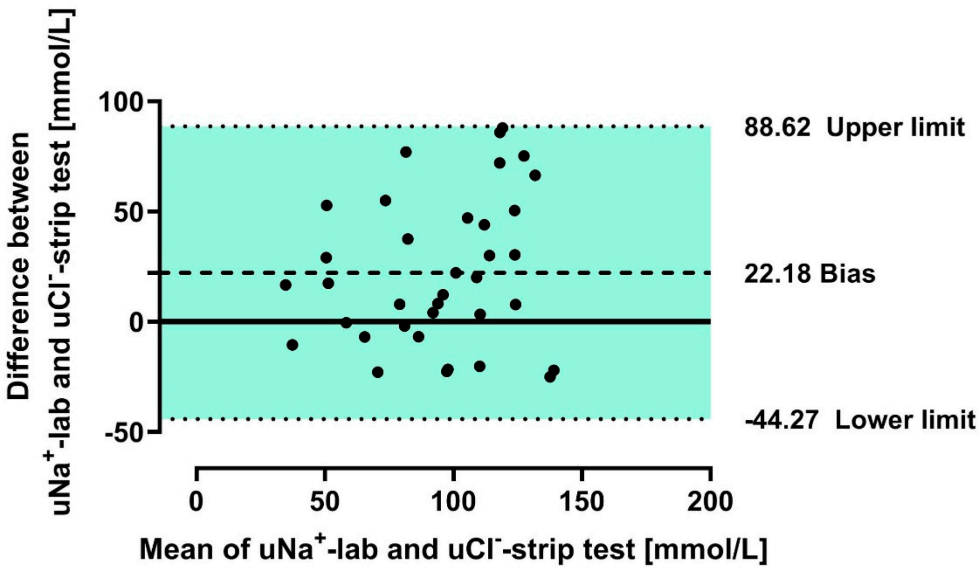

Supplementary Figure S4. *Bland-Altmann plot for agreement of urine chloride concentration measured by strip-test and urine sodium measured in the laboratory in patients with no HF. Legend: uCl^- - urine chloride, uNa^+ - urine sodium, Bias – mean difference between strip test and laboratory test results, Lower limit – mean difference-1.96 × standard deviation, Upper limit – mean difference+1.96 × standard deviation.*

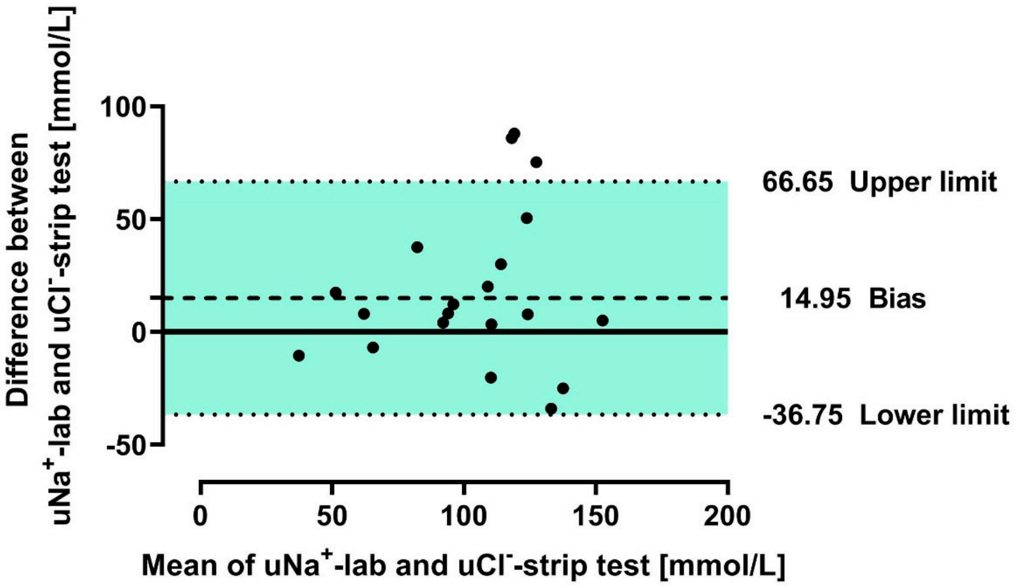

Supplementary Table S2. *Comparison between patients with low and high-eGFR.*

Parameter	Value		p
	**eGFR≤60 ml/min/1.73m2 (N=26)**	**eGFR>60 ml/min/1.73m2 (N=40)**	
Age (years)	71±10	65±13	0.08
Sex – male (N;%)	22 (85%)	30 (75%)	0.25
HR (beats/min)	75 [67-82]	70 [60-80]	0.17
SBP (mmHg)	123±19	123±18	0.27
DBP (mmHg)	75±11	74±11	0.90
MAP (mmHg)	91±13	91±11	0.66
Hgb (g/dl)	11.7±2.1	13.4±1.5	0.01
Leukocytes (10^3/ul)	8.42 [6.76-10.45]	6.72 [5.61-9.02]	0.04
Creatinine (mg/dl)	1.77 [1.46-1.95]	0.89 [0.74-1.07]	<0.001
eGFR (ml/min/1,73m^2)	40±14	91±21	<0.001
Urea (mg/dl)	79 [51-102]	37 [28-46]	<0.001
NT-proBNP (pg/ml)	5649 [2480-14134]	591 [196-2172]	<0.001
Serum Na^+ (mmol/l)	140±2.9	141±2.8	0.21
Serum K^+ (mmol/l)	4.45±0.57	4.36±0.45	0.73
LVEF (%)	33 [28-48]	54 [36-60]	<0.01
TAPSE (mm)	19±4	22±6	0.03
Arterial hypertension (N;%)	23 (88%)	26 (65%)	0.03
Diabetes Mellitus (N;%)	18 (69%)	17 (43%)	0.07
Amyloidosis (confirmed or suspected) (N;%)	1 (4%)	1 (3%)	0.76
Chronic diuretics administration (N;%)	21 (81%)	13 (33%)	<0.001
Chronically administered	60 [40-80]	0 [0-30]	<0.001

loop diuretic dose (furosemide dose equivalent) (mg)			
SGLT-2 inhibitors (N;%)	16 (62%)	17 (43%)	0.13
B-blocker (N;%)	24 (92%)	31 (78%)	0.11
ACEI/ARB (N;%)	9 (35%)	22 (55%)	0.07
ARNI (N;%)	4 (15%)	4 (10%)	0.51
MRA (N;%)	11 (42%)	20 (50%)	0.76
Acetazolamide (N;%)	0 (0%)	0 (0%)	1.00
Thiazide diuretics (N;%)	1 (4%)	2 (5%)	0.54

Supplementary Figure S5. *Bland-Altmann plot for agreement of urine chloride concentration measured by strip-test and urine chloride measured in laboratory in patients with eGFR >60ml/min/1.73m^2. Legend: uCl$^-$ - urine chloride, Bias – mean difference between strip test and laboratory test results, Lower limit – mean difference-1.96 × standard deviation, Upper limit – mean difference+1.96 × standard deviation.*

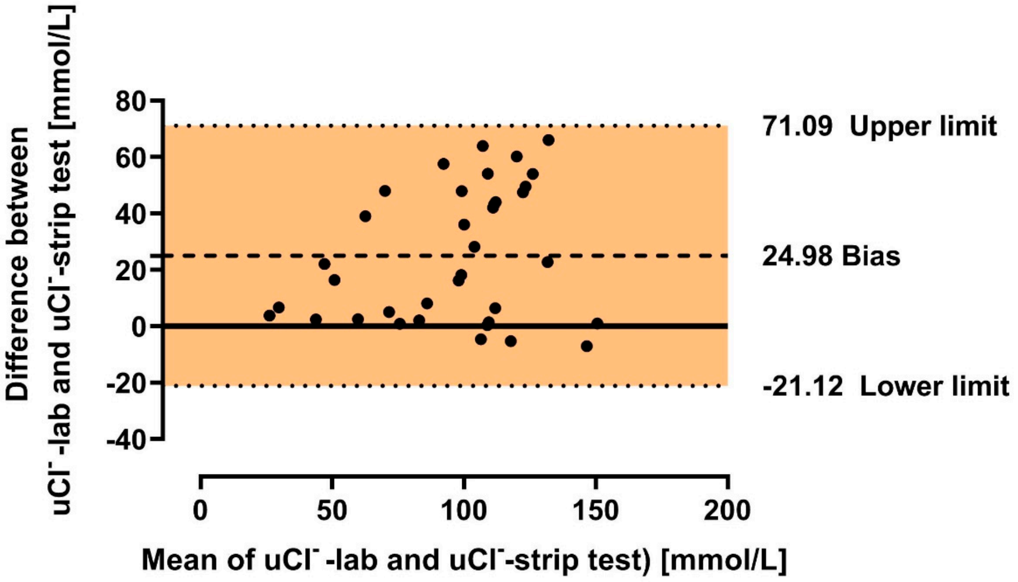

Supplementary Figure S6. *Bland-Altmann plot for agreement of urine chloride concentration measured by strip-test and urine chloride measured in laboratory in patients with eGFR ≤60ml/min/1.73m^2. Legend: uCl$^-$ - urine chloride, Bias – mean difference between strip test and laboratory test results, Lower limit – mean difference-1.96*standard deviation, Upper limit – mean difference+1.96 × standard deviation.*

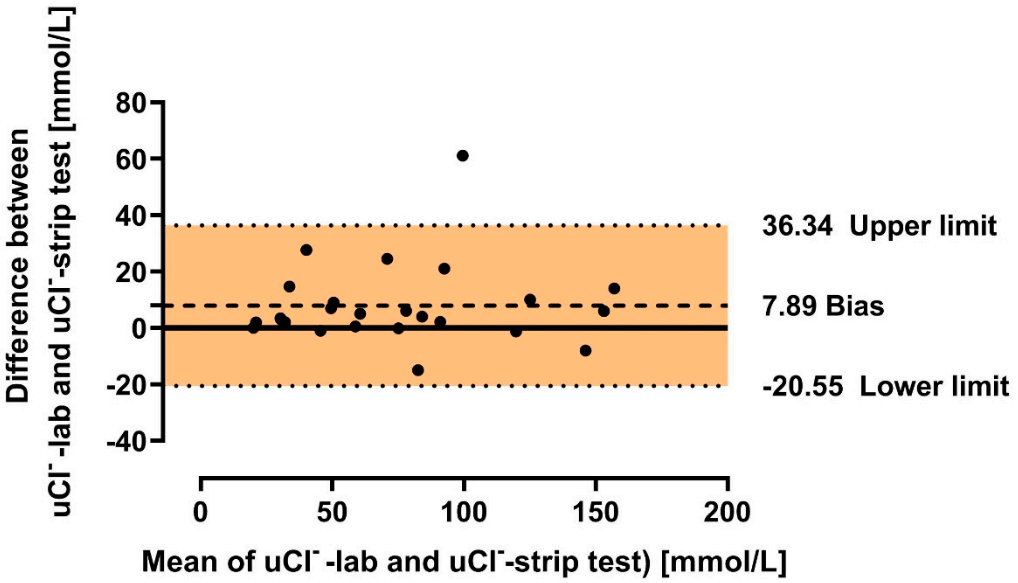

Supplementary Figure S7. *Bland-Altmann plot for agreement of urine chloride concentration measured by strip-test and urine sodium measured in laboratory in patients with eGFR >60ml/min/1.73m². Legend: uCl⁻ - urine chloride, uNa⁺ - urine sodium, Bias – mean difference between strip test and laboratory test results, Lower limit – mean difference-1.96 × standard deviation, Upper limit – mean difference+1.96 × standard deviation.*

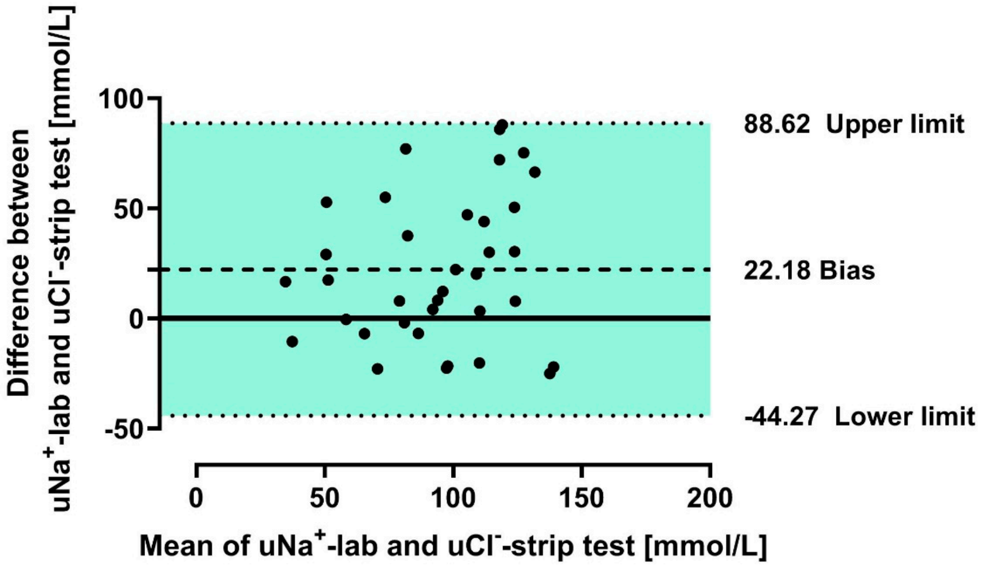

Supplementary Figure S8. *Bland-Altmann plot for agreement of urine chloride concentration measured by strip-test and urine sodium measured in laboratory in patients with eGFR ≤60ml/min/1.73m². Legend: uCl⁻ - urine chloride, uNa⁺ - urine sodium, Bias – mean difference between strip test and laboratory test results, Lower limit – mean difference-1.96 × standard deviation, Upper limit – mean difference+1.96 × standard deviation.*

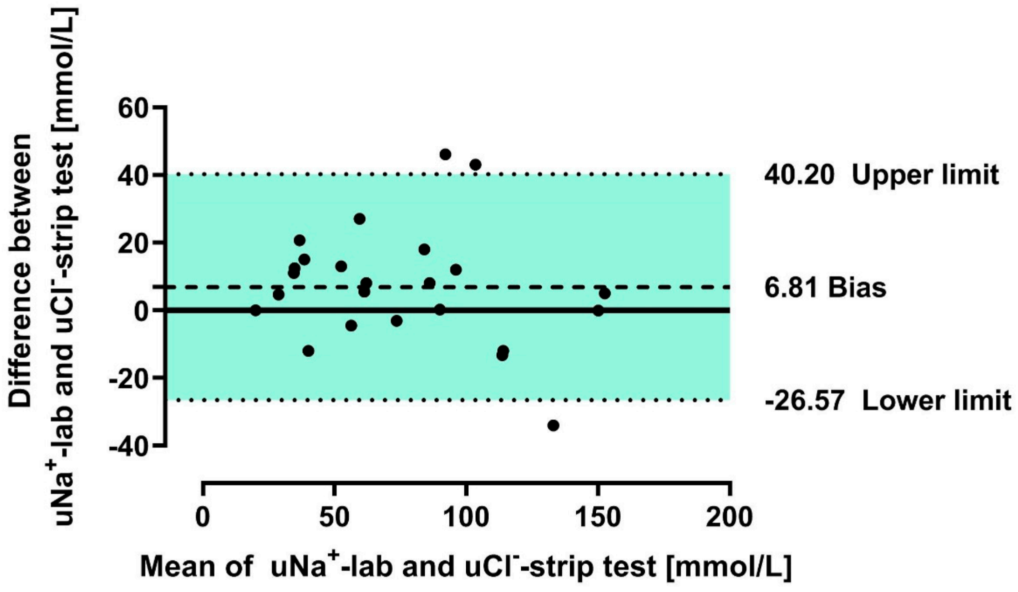

Supplement: Supplementary file 1 [file biomedicines-12-02473-s001.zip › biomedicines-3206765-supplementary.pdf]
